# Public Awareness of Health-Related Risks From Uncontrolled Hypertension

**DOI:** 10.5888/pcd15.170362

**Published:** 2018-04-05

**Authors:** Rahoul Ahuja, Carma Ayala, Xin Tong, Hilary K. Wall, Jing Fang

**Affiliations:** 1 Centers for Disease Control and Prevention, National Center for Chronic Disease Prevention and Health Promotion, Division for Heart Disease and Stroke Prevention, Atlanta, Georgia; 2 Emory University School of Medicine, Department of Internal Medicine, Atlanta, Georgia

## Abstract

Uncontrolled hypertension, a common disorder, is associated with increased long-term risk of several serious conditions. Awareness of the health risks of uncontrolled hypertension is not well understood. We used data from a nationwide panel survey to assess the awareness of risk associated with uncontrolled hypertension, stratified by cardiovascular disease risk factors. Awareness of increased risk from uncontrolled hypertension was high for some outcomes (heart attack, heart failure, stroke), and low for others (kidney disease, dementia). Several disparities in awareness were found. Complementary clinical and public health interventions could be instituted to increase awareness and target people who are high risk.

## Objective

Uncontrolled hypertension increases the risk of heart attack, heart failure, kidney disease, stroke, and cognitive decline (1–3). Low control rates are partly explained by lack of awareness of hypertension; improving awareness could lead to improved control rates (4,5). However, few reports exist on what proportion of adults are aware of health risks from uncontrolled hypertension. We analyzed data from a national, web-based panel survey to assess awareness of risk of cardiovascular disease (CVD) and dementia associated with uncontrolled hypertension stratified by comorbidities, sociodemographic variables, and CVD risk factors.

## Methods

HealthStyles is a web-based panel survey developed and licensed by Porter Novelli Public Services and conducted by GfK’s KnowledgePanel (www.gfk.com/products-a-z/us/knowledgepanel-united-states/) as a probability-based online panel representative of the US adult population aged 18 or older. The survey was sent to 6,166 respondents in June and July of 2016; 4,203 completed the survey (68% response rate). The survey collected data on demographics, health conditions, health beliefs and awareness of health risks, and behaviors. Data were weighted for sex, age, household income, race/ethnicity, household size, education, census region, metropolitan status, and prior Internet access on the basis of the 2015 US Current Population Survey. All data were self-reported. Licensed data were provided; data did not include individual identifiers, was provided in aggregate form, and was granted an exemption by the Centers for Disease Control and Prevention institutional review board.

Primary variables were self-reported hypertension (classified as hypertensive or normotensive) and awareness of increased risk associated with uncontrolled hypertension for serious disorders (heart attack, heart failure, kidney disease, stroke, and dementia). Risk awareness was characterized by asking, “Which, if any, of the following are risks of uncontrolled blood pressure?” Answer choices were heart attack, heart failure, kidney disease, stroke, dementia, and none of these. Characteristics examined included: sex, age (18–44 y, 45–64 y, or ≥65 y), race/ethnicity (non-Hispanic white, non-Hispanic black, non-Hispanic other, or Hispanic), education (less than high school graduate, high school graduate, some college, or college graduate or more), annual household income (<$25,000, $25,000–$39,999, $40,000–$59,999, or ≥$60,000), body mass index (BMI, kg/m^2^) (<25.0 = normal, 25.0–29.9 = overweight, ≥30 = obese), and CVD risk factors (current smoking, obesity), or comorbidities (atrial fibrillation, depression, heart failure, migraine, diabetes, stroke).

We excluded 37 pregnant hypertensive respondents, and the final sample was 4,166. We used Wald χ^2^ tests to assess differences in awareness among hypertensive adults by selected characteristics. Two-tailed *P *values of ≤ .05 were considered significant. All analyses were conducted using SAS version 9.3 (SAS Institute Inc).

## Results

The prevalence of self-reported hypertension was 30.0% (Table 1), with significant differences by age, race/ethnicity, annual household income, education, BMI, and selected comorbidities. Hypertensive adults had significantly higher estimates of awareness for increased risk for stroke associated with uncontrolled hypertension compared with their normotensive counterparts (90.9% vs 77.2%, *P* < .001). Similar patterns were seen among hypertensive adults versus normotensive adults for awareness of risk of heart attack (88.4% vs 78.7%, *P* < .001), heart failure (71.6% vs 65.8%, *P* = .002), and kidney disease (38.5% vs 24.8%, *P* < .001). Though not significant, awareness for risk of dementia was markedly low in both groups: 8.7% for hypertensive adults and 7.9% for normotensive adults (*P* = .47).

**Table 1:** Weighted Percentage^a^ by Sociodemographic Characteristics, Cardiovascular Disease Risk Factors (CVD), and Awareness of Increased CVD Risks Associated With Uncontrolled Blood Pressure Among Respondents (N = 4,166), HealthStyles, 2016^b^

Variable	Total, N (%)	Self-Reported Hypertension		
		Yes	No	*P* Value^c^
		N (%) [95% CI]		
**Total^d^**	4,166 (100)	1,414 (30.0) [28.3–31.6]	2,752 (70.0) [68.4–71.7]	NA
**Demographic Characteristic**				
**Age, y**				
18–44	1,457 (46.1)	188 (17.3) [14.8–19.9]	1,269 (58.4) [56.2–60.5]	<.001
45–64	954 (19.3)	567 (37.6) [34.7–40.6]	387 (11.4) [10.2–12.7]	
≥65	1,755 (34.7)	659 (45.1) [42.0–48.1]	1,096 (30.2) [28.3–32.1]	
**Sex**				
Male	1,996 (48.7)	727 (49.7) [46.6–52.8]	1,269 (48.2) [46.0–50.5]	.45
Female	2,170 (51.3)	687 (50.3) [47.2–53.4]	1,483 (51.8) [49.5–54.0]	
**Race/ethnicity**				
Non-Hispanic white	3,078 (65.2)	1,037 (64.0) [60.8–67.2]	2,041 (65.7) [63.4–68.1]	<.001
Non-Hispanic black	421 (11.7)	189 (17.3) [14.7–19.8]	232 (9.3) [7.9–10.7]	
Hispanic	466 (15.4)	139 (13.5) [11.1–15.9]	327 (16.2) [14.4–18.1]	
Non-Hispanic other^e^	201 (7.7)	49 (5.2) [3.5–7.0]	152 (8.7) [7.1–10.3]	
**Education**				
<High school graduate	275 (12.1)	114 (14.7) [12.0–17.4]	161 (11.0) [9.2–12.7]	<.001
High school graduate	1,238 (29.8)	498 (34.7) [31.8–37.6]	740 (27.6) [25.6–29.7]	
Some college	1,260 (28.4)	418 (26.7) [24.1–29.3]	842 (29.1) [27.1–31.1]	
College graduate or more	1,393 (29.8)	384 (23.9) [21.3–26.4]	1,009 (32.3) [30.3–34.4]	
**Annual household income, $**				
<25,000	709 (17.4)	303 (22.4) [19.7–25.1]	406 (15.2) [13.5–16.9]	<.001
25,000–39,999	670 (13.1)	256 (15.1) [13.1–17.2]	414 (12.2) [10.8–13.6]	
40,000–59,999	683 (15.9)	236 (16.1) [13.9–18.3]	447 (15.8) [14.1–17.4]	
≥60,000	2,104 (53.7)	619 (46.4) [43.3–49.4]	1,485 (56.8) [54.5–59.0]	
**BMI, kg/m^2^ (N = 4,080)**				
<25.0 (normal)	1,335 (35.8)	286 (22.1) [19.4–24.7]	1,049 (41.6) [39.4–43.9]	<.001
25.0–29.9 (overweight)	1,377 (33.0)	453 (32.4) [29.5–35.3]	924 (33.2) [31.1–35.4]	
≥30.0 (obese)	1,368 (31.2)	638 (45.5) [42.4–48.6]	730 (25.2) [23.2–27.1]	
**Comorbidities**				
**Current smoker** **(N = 4,151)**				
Yes	507 (12.4)	165 (13.0) [10.8–15.2]	342 (12.1) [10.6–13.5]	.51
No	3,644 (87.6)	1,245 (87.0) [84.8–89.2]	2,399 (87.9) [86.5–89.4]	
**Diabetes (N = 4,133)**				
Yes	446 (10.0)	314 (22.7) [20.1–25.3]	132 (4.6) [3.6–5.5]	<.001
No	3,687 (90.0)	1,095 (77.3) [74.7–79.9]	2,592 (95.4) [94.5–96.4]	
**High cholesterol (N = 4,133)**				
Yes	897 (18.9)	617 (42.0) [38.9–45.0]	280 (9.0) [7.8–10.3]	<.001
No	3,236 (81.1)	792 (58.0) [55.0–61.1]	2,444 (91.0) [89.7–92.2]	
**Congestive heart failure (N = 4,133)**				
Yes	43 (1.0)	32 (2.6) [1.5–3.7]	11 (0.3) [0.1–0.5]	<.001
No	4,090 (99.0)	1,377 (97.4) [96.3–98.5]	2,713 (99.7) [99.5–99.9]	
**Any cancer (except skin)** **(N = 4,133)**				
Yes	68 (1.6)	39 (2.8) [1.8–3.9]	29 (1.0) [0.6–1.4]	.001
No	4,065 (98.4)	1,370 (97.2) [96.1–98.2]	2,695 (99.0) [98.6–99.4]	
**Migraines** **(N = 4,133)**				
Yes	342 (8.2)	98 (7.4) [5.7–9.2]	244 (8.5) [7.3–9.8]	.31
No	3,791 (91.8)	1,311 (92.6) [90.8–94.3]	2,480 (91.5) [90.2–92.7]	
**Atrial fibrillation** **(N = 4,133)**				
Yes	77 (1.5)	55 (3.3) [2.4–4.3]	22 (0.8) [0.4–1.1]	<.001
No	4,056 (98.5)	1,354 (96.7) [95.7–97.6]	2,702 (99.2) [98.9–99.6]	
**Depression (N = 4,133)**				
Yes	577 (13.8)	252 (18.3) [15.9–20.8]	325 (11.8) [10.3–13.3]	<.001
No	3,556 (86.2)	1,157 (81.7) [79.2–84.1]	2,399 (88.2) [86.7–89.7]	
**Multiple comorbidities **				
None	2,090 (53.9)	431 (31.9) [29.0–34.8]	1,659 (63.3) [61.1–65.4]	<.001
1	1,347 (30.3)	557 (37.4) [34.4–40.3]	790 (27.3) [25.3–29.3]	
≥2	681 (15.8)	417 (30.7) [27.8–33.6]	264 (9.4) [8.1–10.7]	
**Awareness of conditions associated with uncontrolled hypertension (N = 4,129)**				
Heart attack	3,461 (81.7)	1,250 (88.4) [86.4–90.4]	2,211 (78.7) [76.8–80.7]	<.001
Heart failure	2,851 (67.6)	1,021 (71.6) [68.8–74.4]	1,830 (65.8) [63.7–68.0]	.002
Kidney disease	1,208 (28.9)	543 (38.5) [35.5–41.6]	665 (24.8) [22.8–26.8]	<.001
Stroke	3,481 (81.3)	1,299 (90.9) [89.0–92.8]	2,182 (77.2) [75.2–79.2]	<.001
Dementia	336 (8.2)	123 (8.7) [7.0–10.5]	213 (7.9) [6.6–9.2]	.47

Abbreviation: BMI, body mass index.

a Data were weighted for sex, age, annual household income, race/ethnicity, household size, education, census region, metropolitan status, and prior Internet access based on 2015 US Current Population Survey. Of the 4,203 respondents, 37 pregnant women with hypertension were excluded.

b HealthStyles is a web-based panel survey developed and licensed by Porter Novelli Public Services and conducted by GfK’s KnowledgePanel.

c *P* values calculated by using Wald χ^2^ test.

d Total number of respondents with self-reported hypertension who answered the question.

e The overall non-Hispanic other group sample consisted of 24 American Indian/Alaska Natives, 111 Asians, and 101 people of other races. The hypertensive non-Hispanic other group sample consisted of 3 American Indian/Alaska Natives, 25 Asians, and 24 people of other races, sample sizes that were too small to use as individual groups for analyses.

Among hypertensive adults, men were significantly more aware of increased risk for heart attack and heart failure associated with uncontrolled hypertension than women (93.0% vs 83.9%, *P* < .001, and 76.2% vs 67.0%, *P* .001, respectively; Table 2). Compared with adults with lower annual household income, adults with higher household incomes had higher risk awareness estimates of for stroke, kidney disease and dementia. Non-Hispanic whites had higher risk awareness estimates than non-Hispanic blacks for heart attack (90.9% vs 80.7%, *P* = .02), heart failure (74.4% vs 62.8%, *P* = .03), stroke (93.7% vs 87.5%, *P* = .01), and dementia (9.7 vs 5.9, *P* = .01).

**Table 2:** Weighted Percentage^a^ of Awareness of Health Risks Associated with Uncontrolled Hypertension Among Respondents Who Self-Reported Hypertension (N = 1,408), by Selected Characteristics, HealthStyles^b^, 2016

Characteristic	Heart Attack		Heart Failure		Kidney Disease		Stroke		Dementia	
	% (95% CI)	*P* Value	% (95% CI)	*P* Value	% (95% CI)	*P* Value	% (95% CI)	*P* Value	% (95% CI)	*P* Value^c^
Total	88.4 (86.4–90.4)	NA	71.6 (68.8–74.4)	NA	38.5 (35.5–41.6)	NA	90.9 (89.0–92.8)	NA	8.7 (7.0–10.5)	NA
Demographic Characteristic										
Age, y										
18–44	90.4 (85.5–95.3)	.61	73.6 (66.2–81.0)	.52	34.3 (26.4–42.2)	.40	84.2 (77.6–90.8)	.05	5.2 (1.7–8.7)	.12
45–64	88.6 (85.4–91.8)		69.5 (64.8–74.1)		40.6 (35.8–45.5)		93.1 (90.6–95.5)		9.2 (6.1–12.2)	
≥65	87.5 (84.6–90.5)		72.6 (68.6–76.6)		38.4 (34.1–42.8)		91.7 (89.0–94.4)		9.7 (7.1–12.3)	
Sex										
Male	93.0 (91.0–95.0)	<.001	76.2 (72.5–79.9)	.001	39.5 (35.3–43.7)		91.2 (88.4–93.9)	.79	7.8 (5.6–10.1)	.33
Female	83.9 (80.5–87.3)		67.0 (62.8–71.3)		37.6 (33.3–42.0)	.55	90.7 (88.0–93.3)		9.6 (6.9–12.3)	
Race/ethnicity										
Non-Hispanic white	90.9 (89.0–92.9)	.02	74.4 (71.3–77.5)	.03	37.1 (33.8–40.4)	.69	93.7 (91.9–95.4)	.01	9.7 (7.6–11.8)	.01
Non-Hispanic black	80.7 (74.2–87.2)		62.8 (54.8–70.8)		40.7 (32.4–49.0)		87.5 (81.3–93.7)		5.9 (2.1–9.8)	
Hispanic	87.5 (80.9–94.1)		66.9 (57.8–75.9)		40.3 (30.7–50.0)		86.3 (79.4–93.2)		4.0 (0.9–7.0)	
Non-Hispanic other^d^	85.5 (74.2–96.8)		77.7 (63.3–92.1)		44.8 (27.4–62.2)		80.7 (68.3–93.2)		18.0 (3.1–32.8)	
Education										
<High school graduate	88.1 (81.6–94.7)	.60	61.8 (51.6–71.9)	.06	36.9 (26.7–47.2)	.001	84.9 (77.2–92.5)	.23	3.7 (0.2–7.2)	.005
High school graduate	87.2 (83.8–90.7)		70.3 (65.7–74.9)		34.0 (29.3–38.7)		90.7 (87.6–93.9)		7.6 (4.9–10.3)	
Some college	88.3 (84.7–91.9)		74.9 (70.0–79.8)		35.2 (30.0–40.4)		92.7 (89.6–95.8)		7.4 (4.7–10.1)	
College graduate or more	90.5 (87.1–94.0)		75.7 (70.8–80.6)		49.8 (43.8–55.8)		92.9 (89.9–95.9)		14.8 (10.0–19.6)	
Annual household income, $										
<25,000	85.1 (79.8–90.4)	.07	67.0 (60.1–73.8)	.19	27.8 (21.8–33.9)	.001	84.8 (79.4–90.2)	.03	4.9 (2.1–7.8)	.04
25,000–39,999	89.5 (85.6–93.3)		69.1 (62.1–76.1)		37.5 (30.0–44.9)		92.4 (88.6–96.1)		7.1 (3.6–10.6)	
40,000–59,999	84.5 (78.9–90.1)		71.2 (64.5–77.9)		40.8 (33.4–48.2)		90.4 (85.2–95.7)		10.2 (5.7–14.7)	
≥60,000	91.1 (88.6–93.6)		67.0 (60.1–73.8)		43.2 (38.7–47.8)		93.5 (91.2–95.9)		10.5 (7.6–13.5)	
BMI, kg/m^2^										
<25.0 (normal)	83.4 (77.9–88.9)	.09	68.7 (62.2–75.1)		36.0 (29.5–42.4)	.34	88.9 (84.3–93.5)		9.3 (5.7–12.8)	.79
25.0–29.9 (overweight)	89.0 (85.7–92.3)		71.1 (66.0–76.3)		36.1 (30.8–41.4)		91.7 (88.5–94.9)		8.9 (5.5–12.4)	
≥30.0 (obese)	90.4 (87.8–93.0)		72.9 (68.9–76.9)	.55	40.6 (36.1–45.2)		91.2 (88.3–94.1)	.60	7.9 (5.4–10.4)	
Comorbidities										
Current smoker										
Yes	89.9 (84.2–95.7)	.57	72.8 (64.1–81.4)	.76	27.7 (19.8–35.7)	.007	91.0 (85.1–97.0)	>.99	10.1 (5.3–14.9)	.55
No	88.1 (86.0–90.3)		71.3 (68.4–74.3)		40.0 (36.8–43.2)		91.0 (88.9–93.0)		8.6 (6.7–10.5)	
Diabetes										
Yes	88.4 (84.1–92.7)	.98	72.7 (66.7–78.7)	.65	42.9 (36.2–49.5)	.15	90.4 (86.1–94.7)	.79	9.1 (5.1–13.1)	.87
No	88.4 (86.1–90.6)		71.2 (68.0–74.4)		37.3 (33.9–40.7)		91.0 (88.9–93.2)		8.7 (6.7–10.6)	
High cholesterol										
Yes	89.9 (87.0–92.8)	.20	73.7 (69.6–77.8)	.20	40.3 (35.7–44.9)	.34	94.1 (91.8–96.3)	.004	8.8 (6.0–11.7)	.95
No	87.3 (84.5–90.0)		70.0 (66.1–73.8)		37.3 (33.3–41.3)		88.6 (85.7–91.5)		8.7 (6.5–11.0)	
Congestive heart failure										
Yes	100.0	NA^e^	93.7 (86.9–100)	NA^e^	65.7 (46.5–84.8)	.02	98.2 (94.6–100.0)	NA^c^	29.5 (8.8–50.3)	.08
No	88.0 (86.0–90.1)		70.9 (68.1–73.8)		37.8 (34.8–40.9)		90.7 (88.7–92.7)		8.2 (6.5–9.9)	
Any cancer (excluding skin cancer)										
Yes	90.9 (80.4–100.0)	NA^e^	84.0 (72.9–95.1)	.06	25.5 (12.0–39.1)	.10	100.0	NA^c^	12.8 (0.0–27.2)	NA^e^
No	88.3 (86.3–90.3)		71.2 (68.3–74.0)		39.0 (35.9–42.0)		90.6 (88.6–92.6)		8.7 (6.9–10.4)	
Migraine										
Yes	90.9 (84.5–97.4)	.43	71.5 (59.4–83.5)		34.6 (23.0–46.2)		95.2 (91.2–99.2)		11.1 (4.4–17.9)	.47
No	88.2 (86.1–90.3)		71.5 (68.6–74.4)	.99	38.9 (35.8–42.0)	.49	90.5 (88.5–92.6)	.05	8.6 (6.7–10.4)	
Atrial fibrillation										
Yes	96.7 (92.8–100.0)	NA^e^	82.7 (71.9–93.4)	.05	57.9 (43.5–72.2)	.01	97.7 (94.4–100.0)	NA^c^	22.5 (10.1–34.8)	.04
No	88.1 (86.0–90.1)		71.1 (68.2–74.0)		37.9 (34.8–41.0)		90.7 (88.7–92.7)		8.3 (6.5–10.1)	
Depression										
Yes	91.2 (87.0–95.3)	.16	79.2 (72.8–85.6)	.01	34.1 (27.1–41.1)	.17	92.2 (88.1–96.3)	.49	10.2 (6.0–14.3)	.47
No	87.7 (85.5–90.0)		69.8 (66.7–73.0)		39.6 (36.2–42.9)		90.6 (88.4–92.8)		8.5 (6.5–10.4)	
Number of comorbidities^f^										
None	87.7 (84.1–91.2)	.04	70.6 (65.6–75.6)	.002	41.6 (36.1–47.1)	.06	88.7 (84.7–92.6)	.15	7.7 (4.7–10.8)	.29
1	85.9 (82.3–89.6)		66.4 (61.6–71.1)		33.8 (29.2–38.4)		90.9 (87.8–93.9)		7.9 (5.2–10.5)	
≥2	91.8 (88.8–94.9)		78.5 (73.7–83.4)		40.8 (35.1–46.4)		93.4 (90.5–96.3)		11.1 (7.5–14.7)	

Abbreviations: CI, confidence interval; NA, not applicable.

a Data were weighted for sex, age, annual household income, race/ethnicity, household size, education, census region, metropolitan status, and prior Internet access based on 2015 US Current Population Survey. All data were self-reported. Of 4,203 respondents, only 1,408 reported being aware of the risks associated with hypertension.

b HealthStyles is a web-based panel survey developed and licensed by Porter Novelli Public Services and conducted by GfK’s KnowledgePanel.

c *P* values calculated by using Wald χ^2^ test.

d The overall non-Hispanic other group sample consisted of 24 American Indian/Alaska Natives, 111 Asians, and 101 peopole of other races. The hypertensive non-Hispanic other group sample consisted of 3 American Indian/Alaska Natives, 25 Asians, and 24 people of other races; these sample sizes were too small to use as individual groups for analyses.

e Sample size was too small for reliable results.

f Comorbidities were heart attack, heart failure, kidney disease, stroke, and dementia.

Among hypertensive adults, respondents with depression were more likely to be aware of the risk of heart failure from uncontrolled hypertension than those without depression (79.2% vs 69.8%, *P* = .01). Respondents with diabetes had no differences in awareness for all CVD outcomes compared with those without diabetes. Respondents with high cholesterol were more aware of the risks from uncontrolled hypertension for stroke than those without (94.1% vs 88.6%, *P* = .004). Respondents with 2 or more comorbidities had significantly higher risk awareness of heart attack and heart failure associated with uncontrolled hypertension than those with none or one of the conditions (Table 2).

## Discussion

While awareness was high among most respondents for traditionally well-known CVD outcomes (heart attack, heart failure, stroke), it was remarkably lower for others (kidney disease, dementia), suggesting areas for focusing education. As previously reported (6,7), we found notable socioeconomic and racial/ethnic differences in awareness of the risks of hypertension. For example, we found greater awareness of the association between uncontrolled hypertension and kidney disease, stroke, and dementia in high-income households than in low-income households. Non-Hispanic whites had greater risk awareness than non-Hispanic blacks, who have higher prevalence of hypertension and uncontrolled hypertension (8).

Education levels are associated with greater health literacy (7), which influences a patient’s ability to understand hypertension education, complications from uncontrolled hypertension, and disparities in access to hypertension care (9). Increasing access to appropriate clinical care, supported by broad public health initiatives such as promoting use of community health workers to educate patients may help address the lack of awareness of risks from uncontrolled hypertension (7). Moreover, encouraging health care providers to more effectively communicate the dangers of uncontrolled hypertension can improve adherence to medications and prognosis (10). Additionally, risk awareness initiatives should tailor interventions to people with both diabetes and hypertension given the concurrent and heightened CVD and chronic kidney disease risks uncontrolled hypertension poses for them.

Initiatives — such as the National Institute of Neurological Disorders and Stroke’s Mind Your Risks (https://mindyourrisks.nih.gov/), World Kidney Day (www.worldkidneyday.org/), Million Hearts (https://millionhearts.hhs.gov/), Measure Up/Pressure Down (www.measureuppressuredown.com/), and Target BP (http://news.heart.org/aha-ama-launch-high-blood-pressure-initiative/) — should be expanded to reach more diverse populations to further increase awareness of the detrimental effects of uncontrolled hypertension (ie, impaired cognitive and kidney function).

Our study’s limitation was that the survey was subject to recall bias, which is inherent in self-reported data and convenience sampling. The strength of our study is that the panel was drawn from a quota sampling to obtain a study group that matched the US population on 7 census demographics. 

This study highlights the areas of need for educating populations who are unaware of the risks from uncontrolled hypertension. With nearly 35 million people with uncontrolled hypertension (4), effective interventions are needed to raise awareness of its risks. Public health initiatives should be replicated and expanded for more diverse populations to provide education on the risks of uncontrolled hypertension.
